# Corynebacterium drakensteinense sp. nov., isolated from the nasopharynx of a healthy South African infant

**DOI:** 10.1099/ijsem.0.007068

**Published:** 2026-02-05

**Authors:** Robbie R. Haines, Anastasia Basuki, Vanessa P. Tenaglia, Heather J. Zar, Mark P. Nicol, Ritika Kar Bahal

**Affiliations:** 1 The Marshall Centre for Infectious Diseases Research and Training, The University of Western Australia, Nedlands, WA, Australia; 2 School of Biomedical Sciences, The University of Western Australia, Nedlands, WA, Australia; 3 Wesfarmers Centre for Vaccines and Infectious Diseases, The Kids Institute, Nedlands, WA, Australia; 4 Department of Paediatrics and Child Health, University of Cape Town, Cape Town, South Africa

**Keywords:** *Actinomycetota*, commensal, *Corynebacterium*, infant, nasopharynx

## Abstract

Emerging evidence supports the role of the nasopharyngeal microbiome in respiratory health, including association with conditions such as asthma and respiratory tract infections. One dominant commensal genus is *Corynebacterium*, members of which are commonly present in the nasopharynx of infants. These commensal *Corynebacterium* spp. have been reported to correlate with respiratory health. In this paper, we present isolate MNWGS58^T^ isolated from the nasopharynx of a South African infant. Genomic analysis of the whole-genome sequence of MNWGS58^T^ revealed that it is phylogenetically closely related to other *Corynebacterium* spp. found in the nasopharynx, *Corynebacterium propinquum* [85% average nucleotide identity (ANI)] and *Corynebacterium pseudodiphtheriticum* (84% ANI). Bacterial identification using matrix-assisted laser desorption/ionization time-of-flight MS identified MNWGS58^T^ as *C. pseudodiphtheriticum*. The API Coryne assay identified the novel isolate as *C. propinquum*, and the VITEK 2 ANC assay identified the novel isolate as *Corynebacterium otitidis*. Both genomic analyses and phenotypic analyses show striking similarities to *C. propinquum* and *C. pseudodiphtheriticum*. The cell wall is consistent with closely related *Corynebacterium* spp., albeit with a higher C_17:0_ content. The genome is 2.48Mbp with a G+C content of 56.9 mol%. Digital DNA–DNA hybridization values for MNWGS58^T^ were low when compared to *C. pseudodiphtheriticum* MNWGS56 and *C. propinquum* MNWGS51 (27.4 and 28.4%, respectively). Although there are phenotypic similarities, 85% ANI with the closest *Corynebacterium* spp. strongly supports the classification of a novel species of *Corynebacterium*, for which we propose the name *Corynebacterium drakensteinense* sp. nov., with the type strain MNWGS58^T^ (=TSD-445^T^=NCTC 15058^T^). It will be important to elucidate the role of this novel species of *Corynebacterium* in the human nasopharynx and identify additional niches for this species in future studies.

## Introduction

Several cohort studies provide evidence that *Corynebacterium* spp. are present in the nasopharyngeal microbiome in early childhood, particularly in the first year of life [1,3]. Nasopharyngeal *Corynebacterium* spp. in infants have been associated with significant health benefits, such as protection from recurrent respiratory tract infections [4]. Commensal *Corynebacterium* spp. have been shown to have an inhibitory effect on *Streptococcus pneumoniae*, a known pathobiont of the nasopharynx [5]. Additionally, in a mouse model, *Corynebacterium pseudodiphtheriticum* conferred increased resistance to respiratory syncytial virus infection [6]. The manner in which *Corynebacterium* spp. exert this protective effect is not yet fully understood; however, *in vitro* data support the notion that the secretion of siderophores may deprive pathogens of iron that is essential for their successful colonization [7].

The bacterial composition of the nasopharyngeal microbiome, especially during childhood, is a key determinant of susceptibility to respiratory infections and may influence respiratory health [1,8, 9]. Microbiome studies have relied heavily on 16S rRNA gene amplicon sequencing for determining the microbial composition; however, this approach often fails to distinguish to the species level [10]. For instance, the 16S rRNA gene of common nasopharyngeal *Corynebacterium* spp., *C. pseudodiphtheriticum* and *Corynebacterium propinquum*, is >99% conserved. Whole metagenome shotgun sequencing or single isolate-level sequencing of the microbiome enables species- or strain-level characterization of the microbiome, including the identification of novel species.

This study describes the identification of strain MNWGS58^T^ isolated from the nasopharynx of an infant in South Africa [11]. Based on its biochemical, phylogenetic and genomic characteristics, we propose that this strain be classified as a novel species, *Corynebacterium drakensteinense* sp. nov.

## Methods

### Sample collection and isolation of bacteria

In the Drakenstein Child Health study, flocked nasopharyngeal swabs were collected in skim milk, tryptone, glucose and glycerin (STGG) broth and stored at −80 °C [12]. In this case, the stored swab belonged to a 30-week-old, male, HIV-exposed, but HIV-uninfected. The child did not develop any symptomatic lower respiratory tract infection through 5 years of age. The approximate location of the study participant at the time of sampling was Paarl, Western Cape, South Africa (33° 43′ 48″ S 18° 57′ 36″ E) as part of the Drakenstein Child Health Study [11]. Briefly, 100 µl of STGG samples were spread on Columbia agar base supplemented with 5% sheep’s blood (blood agar) and chocolate agar for cultivation at 37 °C in aerobic conditions enriched with 5% CO_2_ to replicate the conditions in the nasopharynx. After 24–48 h, morphologically distinct colonies were streaked for purity, and pure bacterial cultures were stored in brain-heart infusion broth supplemented with 25% glycerol for long-term storage at −80 °C. Presumptive identities of each bacterial isolate were made using matrix-assisted laser desorption/ionization time-of-flight (MALDI-TOF) MS (MALDI Biotyper sirius one IVD system, Bruker) with the latest sirius one library as of September 2023.

### Next-generation sequencing and genomic characterization

Next-generation sequencing was used to determine genome sequence and confirm identity.

Genomic DNA was extracted from pure culture using Qiagen DNeasy Blood and Tissue kit with enzymatic lysis (20 mM Tris-Cl pH 8.0, 2 mM sodium EDTA, 1.2% Triton X-100 and lysozyme 50 mg ml^−1^). The concentration of the extracted DNA was measured by Qubit^™^ dsDNA Quantification assay kit according to manufacturer’s instructions. DNA was sequenced using an Illumina MiSeq platform in conjunction with the MiSeq Reagent Kit v3 2×300 bp (Illumina, San Diego, CA, USA). Reads were filtered for quality using the bbduk from the BBTools suite with the following settings: qtrim=r trimq=20 ktrim=r k=23 mink=11 hdist=1 tpe=t tbo=t [13]. Quality-controlled reads were introduced into the SPAdes (v3.13.1) assembly pipeline to produce contigs [14]. Contigs <500 bp were removed from the assembly. The DFAST annotation tool (v1.2.14) was used to annotate the draft genome with coding sequences (CDSs), clustered regularly interspaced short palindromic repeats (CRISPRs), tRNA genes and rRNA genes, including the 16S rRNA gene [15]. Functional data on CDS were later annotated using the KEGG orthology.

The National Centre for Biotechnology Information’s (NCBI) basic local alignment search tool (blast) was utilized with the 16S rRNA database to obtain a genus-level identity for the isolate using the previously identified 16S rRNA gene. Once the genus was identified, all genomes of that genus deposited in the NCBI Reference Sequence database were downloaded. Average nucleotide identity (ANI) was calculated using the fastANI tool, with the genome sequence of the isolate used as the query, and the Reference Sequence database of the genus was used as the reference [16]. Digital DNA–DNA hybridization (dDDH) values were calculated using genome-to-genome distance calculator (v3.0) [17]. A phylogenomic tree of all species belonging to the genus was constructed using GToTree (v1.8.2), which was configured to perform multiple sequence alignments of 138 conserved *Actinomycetota* genes using the gene library described in the GToTree tool documentation [18]. Additionally, FastTree was used to generate a phylogenetic tree of the 16S rRNA gene in the genus *Corynebacterium* (bootstrap calculated from 1,000 replicates) [19]. Both SPAdes and GToTree have several dependencies, which can be found in the documentation for these tools [20,25]. Interactive Tree of Life (iTOL) version 7.4 was used to visualize all trees [26].

### Phenotypic characterization

Metabolic and phenotypic characterization was carried out using the VITEK 2 ANC assay (bioMérieux SA, Marcy-l'Étoile, France) and the API Coryne assay (bioMérieux SA, Marcy-l'Étoile, France) following the manufacturer’s instructions. MICs to seven antimicrobial agents (erythromycin, gentamicin, penicillin, vancomycin, ciprofloxacin, meropenem and tetracycline) were determined using the standard antimicrobial susceptibility testing described by the Clinical Laboratory Standards Institute’s protocol for coryneform bacteria [27]. Cell wall fatty acid composition was determined by triple quadrupole MS, and spectrometry peaks were annotated using the Shimadzu Smart Fatty Acids Database.

### Morphology

Photographs of bacterial colonies on blood agar were captured using a dissecting microscope equipped with a digital eyepiece with a calibration device and images had scales added using ImageJ (v1.54g, National Institutes of Health, USA) [28]. Gram-stained slides were prepared and imaged at 1,000× magnification using light microscopy (Olympus BX43) with a digital camera T piece (Olympus DP27), and scale bars were added using Olympus Cellsens entry v2.3. Scanning electron microscopy (Zeiss 1555 VP-FESEM) was used to gain higher resolution images of cell morphology.

## Results

### Genome features

A total of 154 Mbp in 540,672 reads were obtained by Illumina sequencing. Following quality control and processing, 57 Mbp in 536,497 reads remained, resulting in 45.8× technical coverage. A total of 43 contigs were assembled with an N_50_ of 129 kbp. DFAST detected 2,227 CDS, 3 rRNA genes, 47 tRNA genes and 4 CRISPRs. The final assembly for the whole genome of MNWGS58^T^ was determined to be 2.48 Mbp with a G+C content of 56.9 mol%. The 16S rRNA gene sequence was deposited in NCBI GenBank under accession number OR912475. The annotated draft genome was deposited in NCBI Genome as *Corynebacterium* sp. MWGS58 accession number JAXOFP000000000.1.

### Phylogenetic and genomic analysis

**Fig. 1.**
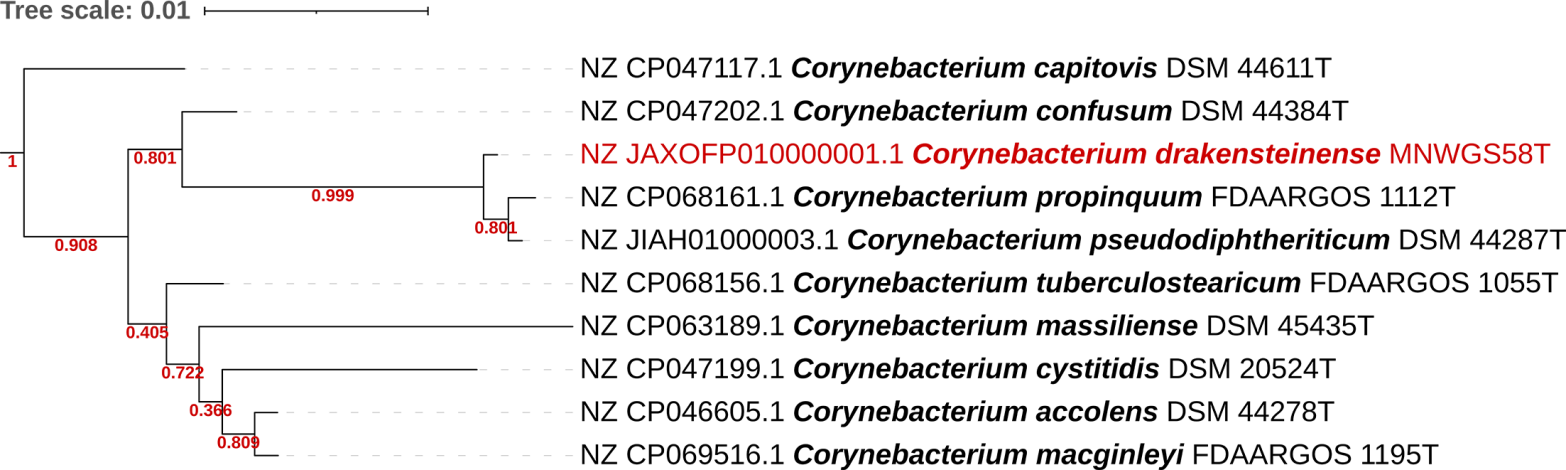
Phylogenetic subtree of the full-length 16S rRNA gene for MNWGS58^T^ (shown in red) and phylogenetically close type strains. Scale bar shows substitutions per site as calculated using the FastTree command line tool and visualized using iTOL. Bootstrap values from 1,000 replicates are shown in red on branches. Accession numbers correspond to the NCBI RefSeq database.

**Fig. 2.**
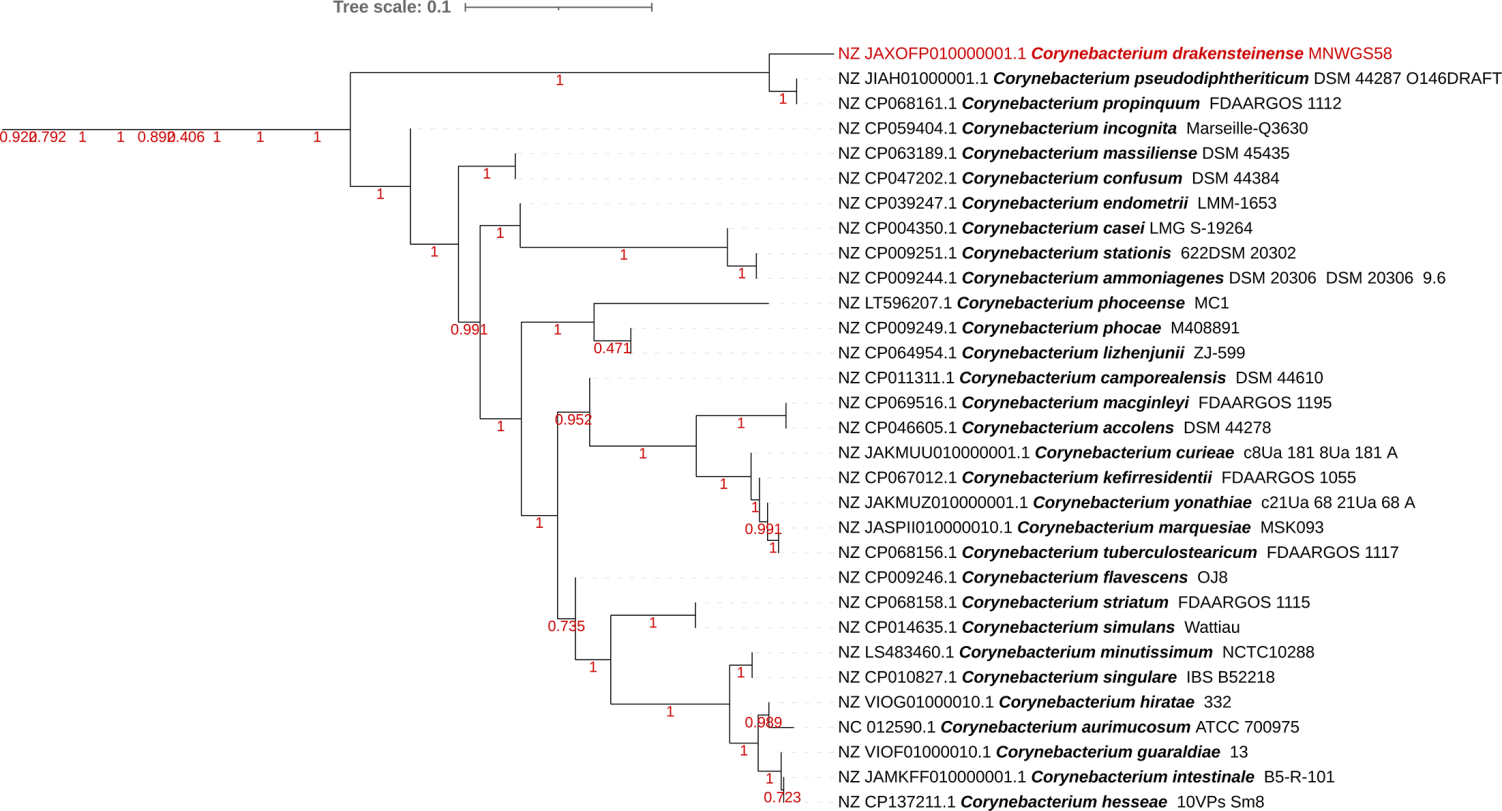
Phylogenomic subtree of the genus *Corynebacterium* evaluated using 138 conserved genes in the phylum *Actinomycetota* using the GToTree tool. Trees were visualized using iTOL. Values shown are arbitrary distance values as calculated by GToTree. Bootstrap values are shown in red. MNWGS58^T^ is also shown in red. Full tree containing all *Corynebacterium* spp. is available as Fig. S1.

Based on the NCBI 16S rRNA gene database, the 16S rRNA gene sequence of MNWGS58^T^ exhibited 99.45% similarity to *C. propinquum* (NCBI accession NR_037038.1) and 99.27% similarity to *C. pseudodiphtheriticum* (NCBI accession NR_042137.1). All 16S rRNA hits with >95% identity to the full-length 16S rRNA gene for MNWGS58^T^ are shown in Table 1. Additionally, a 16S phylogenetic tree can be seen in Fig. 1. For the whole genome, fastANI only returned two matches with an ANI of ≥70% with the full genome sequence of the novel species: *C. propinquum* (85.12% ANI) and *C. pseudodiphtheriticum* (84.24% ANI). The low ANI values indicate that MNWGS58^T^ is a new species, not yet described in the literature. Similarly, dDDH values were low: 28.4 and 27.4% when comparing MNWGS58^T^ to *C. propinquum* MNWGS51 and *C. pseudodiphtheriticum* MNWGS56, respectively. Phylogenomic analysis shows clustering of MNWGS58^T^ with *C. propinquum* MNWGS51 and *C. pseudodiphtheriticum* MNWGS56 (Fig. 2). The full phylogenomic tree is available as Fig. S1, available in the online Supplementary Material. A spreadsheet of KEGG orthologs detected in *C. propinquum* MNWGS51, *C. pseudodiphtheriticum* MNWGS56 and MNWGS58^T^ is contained in Fig. S2.

**Table 1.** Hits of the full-length 16S rRNA gene of MNWGS58^T^ using the 16S rRNA database in the NCBI’s blast Only hits with >95% identity are shown.

Species and strain	Percentage identity	Accession*
*C. propinquum* B 77159	99.45	NR_037038.1
*C. propinquum* DSM 44285 (=CIP 103792)	99.28	NR_119118.1
*C. pseudodiphtheriticum* CIP 103420	99.27	NR_042137.1
*C. pseudodiphtheriticum* NCTC 11136	99.19	NR_119176.1
*Corynebacterium tuberculostearicum* Medalle X	95.53	NR_028975.1
*Corynebacterium macginleyi* JCL-2	95.5	NR_042138.1
*Corynebacterium accolens* CNCTC Th1/57	95.44	NR_042139.1
*Corynebacterium aquilae* S-613	95.31	NR_028989.1
*Corynebacterium confusum* DMMZ 2439	95.25	NR_026449.1
*Corynebacterium minutissimum* NCTC 10288	95.24	NR_037124.1
*Corynebacterium tuberculostearicum* ATCC 35692	95.03	NR_119173.1

* Accession refers to the NCBI accession number

### Phenotypic characteristics

MALDI-TOF MS identified MNWGS58^T^ as *C. pseudodiphtheriticum* with a score of >2.0, indicating a high-confidence identification. This suggests that there is a high degree of overlap between the mass spectrum of the MNWGS58^T^ and the reference mass spectrum of *C. pseudodiphtheriticum* in the MALDI-TOF MS database.

The VITEK 2 ANC (Anaerobic and Coryneform) cards use a panel of 36 biochemical tests to identify a bacterial isolate. Using the VITEK 2, MNWGS58^T^ was a close match (97%) to *Corynebacterium otitidis* (formerly *Turicella otitidis*) (Table 2). The VITEK 2 report also notes that a positive result in the Ala-Phe-Pro arylamidase (APPA) test is not typical for *C. otitidis*, while it is typical of both *C. propinquum* and *C. pseudodiphtheriticum*.

**Table 2.** Phenotypic features of MNWGS58^T^ and closely related species, using the VITEK 2 ANC assay

Biochemical substrate	MNWGS58^T^	*C. pseudodiphtheriticum* MNWGS56	*C. propinquum* MNWGS51
d-Galactose	−	−	−
Leucine arylamidase	+	−	+
Ellman	−	−	−
Phenylalanine arylamidase	+	+	+
l-Proline arylamidase	+	+	+
l-Pyrrolidonyl arylamidase	−	−	−
d-Cellobiose	−	−	−
Tyrosine arylamidase	+	+	+
Ala-Phe-Pro arylamidase	+	+	+
d-Glucose	−	−	−
d-Mannose	−	−	−
d-Maltose	−	−	−
Saccharose/sucrose	−	−	−
Arbutin	−	−	−
*N*-Acetyl-d-glucosamine	−	−	−
5-Bromo-4-chloro-3-indoxyl-*β*-glucoside	−	−	−
Urease	−	+	+
5-Bromo-4-chloro-3-indoxyl-*β*-glucuronide	−	−	−
*β*-Galactopyranosidase indoxyl	−	−	−
*α*-Arabinosidase	−	−	−
5-Bromo-4-chloro-3-indoxyl-*α*-galactoside	−	−	−
*β*-Mannosidase	−	−	−
Arginine GP	−	−	−
Pyruvate	+	+	+
Maltotriose	−	−	−
Aesculin hydrolysis	−	−	−
*β*-d-Fucosidase	−	−	−
5-Bromo-4-chloro-3-indoxyl-*β-N*-acetyl-glucosamide	−	−	−
5-Bromo-4-chloro-3-indoxyl-*α*-mannoside	−	−	−
*α*-l-Fucosidase	−	−	−
Phosphatase	−	−	−
l-Arabinose	−	−	−
d-Ribose 2	−	−	−
Phenylphosphonate	−	−	+
*α*-l-Arabinofuranoside	−	−	−
d-Xylose	−	−	−

The API Coryne assay uses a smaller scale panel of biochemical tests compared to VITEK 2 for identification of coryneform bacteria to species level. The API Coryne assay identified MNWGS58^T^ as *C. propinquum* with an 89.7% probability and a note ‘POSSIBILITY OF *Rhodococcus equi*’. API Coryne results are shown in Table 3. Notably, MNWGS58^T^ is negative for pyrrolidonyl arylamidase (PyrA), while *C. propinquum* and *C. pseudodiphtheriticum* are positive for PyrA. This may be a useful biochemical test for differentiating MNWGS58^T^ from these closely related species. PyrA is involved in the catabolic metabolism of peptides, which some organisms may rely on as a primary carbon or nitrogen source.

**Table 3.** Phenotypic features of MNWGS58^T^ and closely related species, as tested using the API Coryne assay; resultant database profile for MNWGS58^T^ is 1100004

Phenotype tested	MNWGS58^T^	*C. pseudodiphtheriticum* MNWGS56	*C. propinquum* MNWGS51
Reduction of nitrates	+	+	+
Expression of pyrazinamidase	−	−	−
Expression of pyrrolidonyl arylamidase	−	+	+
Expression of alkaline phosphatase	+	+	+
Expression of *β*-glucuronidase	−	−	−
Expression of *β*-galactosidase	−	−	−
Expression of *α*-glucosidase	−	−	−
Expression of *N*-acetyl-*β*-glucosaminidase	−	−	−
Hydrolysis of aesculin	−	−	−
Expression of urease	−	+	−
Hydrolysis of gelatin	−	−	−
Utilization of d-glucose	−	−	−
Utilization of d-ribose	−	−	−
Utilization of d-xylose	−	−	−
Utilization of d-mannitol	−	−	−
Utilization of d-maltose	−	−	−
Utilization of d-lactose	−	−	−
Utilization of d-saccharose	−	−	−
Utilization of glycogen	−	−	−
Expression of catalase	+	+	+

**Fig. 3.**
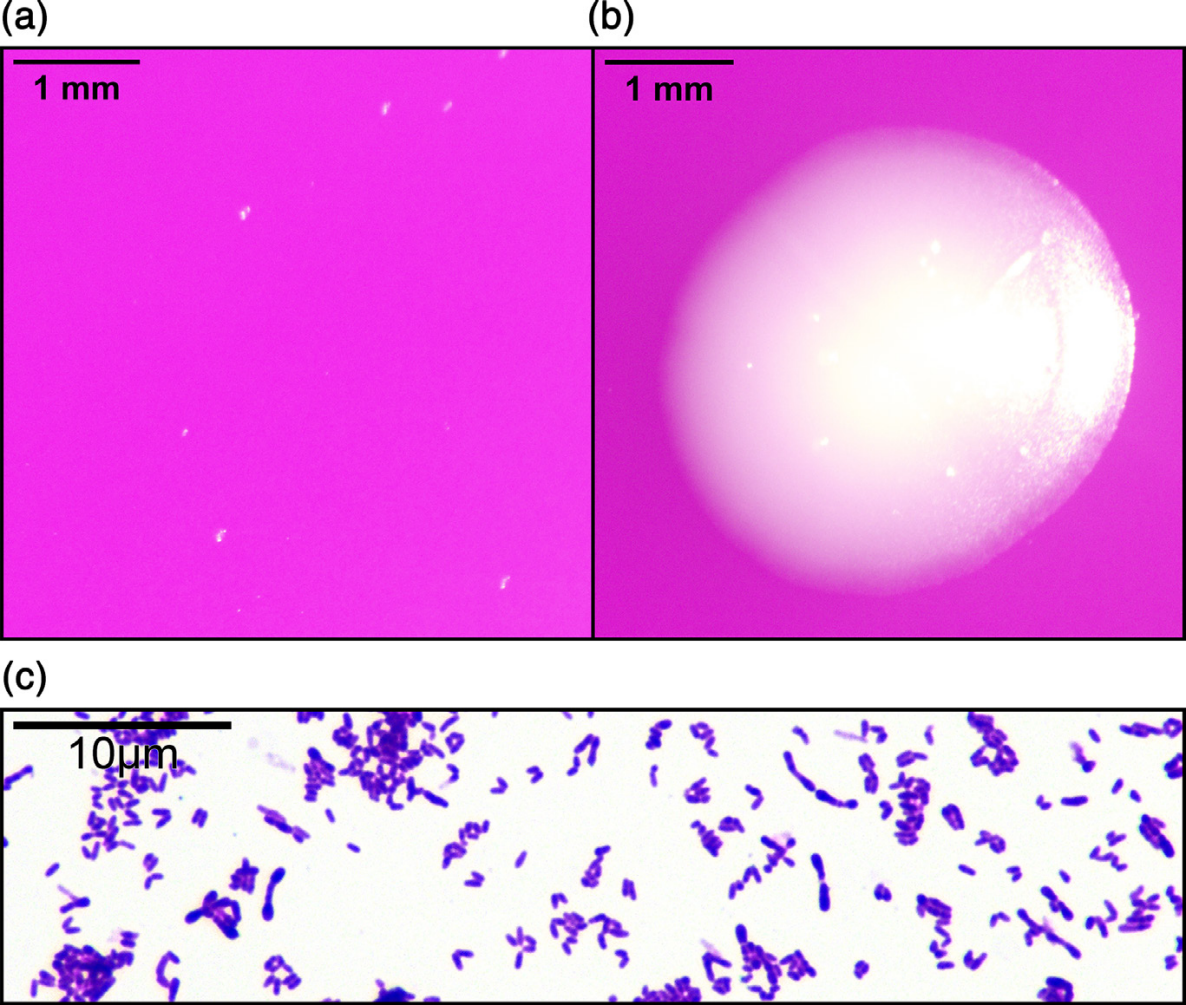
(a) Pinpoint MNWGS58^T^ colonies on blood agar after 24 h of incubation (37 °C, 5% CO2). (b) Mature MNWGS58^T^ colony on blood agar after 48 h of incubation (37 °C, 5% CO2). (c) Gram stain of MNWGS58^T^ showing Gram-positive rod-shaped cells with coryneform morphology.

**Fig. 4.**
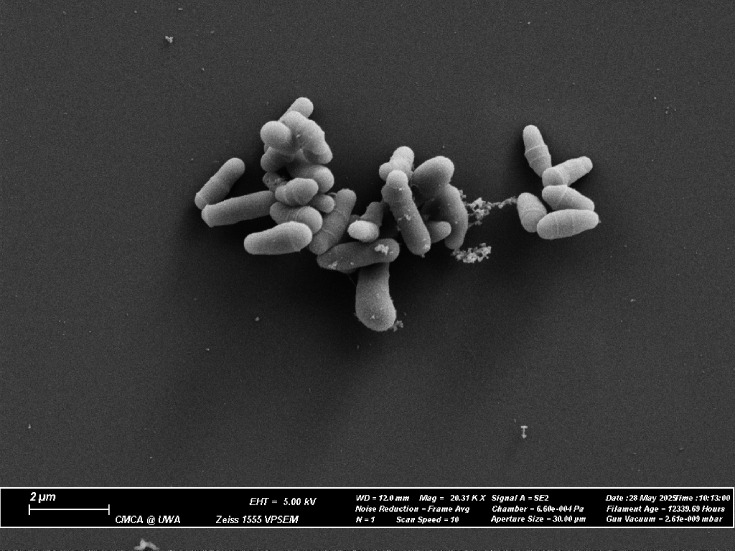
Scanning electron microscope image of MNWGS58^T^.

Fatty acid methyl ester analysis showed that the cell wall of MNWGS58^T^ had a substantially higher amount of C_17:0_ than its phylogenetically close relatives (Table 4). MICs were determined for MNWGS58^T^ and its close relatives MNWGS56 and MNWGS51 against erythromycin, gentamicin, penicillin, vancomycin, ciprofloxacin, meropenem and tetracycline. MNWGS58^T^ was sensitive to all antimicrobials tested. Colony pictures at 24 and 48 h timepoints are shown in Fig. 3, alongside a Gram stain at the 48 h time point. Scanning electron microscopy is shown in Fig. 4. Both optical and scanning electron microscopy show discrete 1–2 µm coryneform cells. The cells are Gram-stain positive, with optimal growth observed after incubation on blood agar for 48 h at 37 °C in aerobic conditions enriched with 5% CO_2_.

**Table 4.** Cell wall fatty acid composition as determined by fatty acid methyl ester analysis using triple quadrupole MS

Fatty acid	Percentage of cell wall fatty acids detected		
	MNWGS58^T^	*C. pseudodiphtheriticum* MNWGS56	*C. propinquum* MNWGS51
Saturated			
C_12:0_	0.06	0.07	0.08
C_14:0_	1.03	0.88	0.65
C_15:0_	0.61	0.07	0.16
C_16:0_	63.13	63.22	42.32
C_17:0_	3.60	0.41	1.03
C_18:0_	22.87	27.80	51.65
C_19:0_	0.10	0.04	0.05
C_20:0_	0.14	0.17	0.25
C_22:0_	0.05	0.06	0.04
C_23:0_	0.02	0.01	0.01
C_24:0_	0.04	0.04	0.02
Unsaturated			
C_16:1_	0.75	0.36	0.20
C_18:1_	7.47	6.78	3.43
C_18:2_	0.08	0.04	0.08
C_20:1_	0.05	0.05	0.03

Given the clear genetic, genomic and phenotypic divergence from other *Corynebacterium* spp., we propose that strain MNWGS58^T^ represents a novel species, for which the name *C. drakensteinense* sp. nov. is proposed. The species name reflects its geographic origin.

## Description of *Corynebacterium drakensteinense* sp. nov.

*Corynebacterium drakensteinense* (dra.ken.stein.en’se. N.L. neut. adj. *drakensteinense*, pertaining to the Drakenstein municipality, South Africa).

Aerobic, Gram-stain positive, non-motile, 1–2 µm rod-shaped cells with typical coryneform clubbing visible by optical and electron microscopy. Optimal growth is observed on blood agar at 37 °C in aerobic conditions enriched with 5% CO_2_ for 48 h. Small colonies, ~1 mm in diameter, appear within 24 h. Mature colonies are circular and 2–4 mm in diameter with well-demarcated sharp margins. The colonies are white, and the centre is slightly raised from the growth medium. Non-CO_2_ enriched incubation yields few stunted colonies. Does not use glucose, mannose, maltose, xylose, arabinose, maltotriose or sucrose as a carbon source. Uses pyruvate as a carbon source. Metabolizes amino acids as a nitrogen and carbon source. Can reduce nitrates and is catalase positive.

The type strain is MNWGS58^T^ (=TSD-445^T^=NCTC 15058^T^), which was isolated from the nasopharynx of a healthy 30-week-old South African child in the Drakenstein municipality. The whole-genome length is 2.48 Mb with 56.9 mol% G+C content.

The annotated genome of strain MNWGS58^T^ is deposited on NCBI Genome with accession number JAXOFP000000000, and the 16S rRNA gene is deposited into NCBI GenBank with accession number OR912475.

## Supplementary material

10.1099/ijsem.0.007068 Uncited Supplementary Material 1.

## Abbreviations

ANI: average nucleotide identity
blast: basic local alignment search tool
CDS: coding sequence
CRISPRs: clustered regularly interspaced short palindromic repeats
dDDH: digital DNA–DNA hybridization
iTOL: Interactive Tree of Life
MALDI-TOF: matrix-assisted laser desorption/ionization time-of-flight
NCBI: National Centre for Biotechnology Information
STGG: skim milk, tryptone, glucose and glycerin
