# Corrigendum: Corynebacterium drakensteinense sp. nov., isolated from the nasopharynx of a healthy South African infant

**DOI:** 10.1099/ijsem.0.007118

**Published:** 2026-03-23

**Authors:** Robbie R. Haines, Anastasia Basuki, Vanessa P. Tenaglia, Heather J. Zar, Mark P. Nicol, Ritika Kar Bahal

**Affiliations:** 1 The Marshall Centre for Infectious Diseases Research and Training, The University of Western Australia, Nedlands, WA, Australia; 2 School of Biomedical Sciences, The University of Western Australia, Nedlands, WA, Australia; 3 Wesfarmers Centre for Vaccines and Infectious Diseases, The Kids Institute, Nedlands, WA, Australia; 4 Department of Paediatrics and Child Health, University of Cape Town, Cape Town, South Africa

In the published version of this article an incorrect culture collection deposit number for the type strain of *Corynebacterium drakensteinense* sp. nov. was provided in the author notes and protologue. Specifically, the National Collection of Type Cultures accession number NCTC 15058 was provided. The correct accession number for the type strain is NCTC 15084.

The incorrect accession number appear in the Abstract, Author Notes, and Protologue. Corrected versions of the Abstract, Author Notes, and Protologue are presented below.

The authors apologise for any inconvenience caused.

The authors would like to acknowledge Dr Seán Turner (National Centre for Biotechnology Information, National Institutes of Health, USA) for informing us of this error.

**Abstract**

Emerging evidence supports the role of the nasopharyngeal microbiome in respiratory health, including association with conditions such as asthma and respiratory tract infections. One dominant commensal genus is *Corynebacterium*, members of which are commonly present in the nasopharynx of infants. These commensal *Corynebacterium* spp. have been reported to correlate with respiratory health. In this paper, we present isolate MNWGS58^T^ isolated from the nasopharynx of a South African infant. Genomic analysis of the whole-genome sequence of MNWGS58^T^ revealed that it is phylogenetically closely related to other *Corynebacterium* spp. found in the nasopharynx, *Corynebacterium propinquum* [85% average nucleotide identity (ANI)] and *Corynebacterium pseudodiphtheriticum* (84% ANI). Bacterial identification using matrix-assisted laser desorption/ionization time-of-flight MS identified MNWGS58^T^ as *C. pseudodiphtheriticum*. The API Coryne assay identified the novel isolate as *C. propinquum*, and the VITEK 2 ANC assay identified the novel isolate as *Corynebacterium otitidis*. Both genomic analyses and phenotypic analyses show striking similarities to *C. propinquum* and *C. pseudodiphtheriticum*. The cell wall is consistent with closely related *Corynebacterium* spp., albeit with a higher C17:0 content. The genome is 2.48Mbp with a G+C content of 56.9 mol%. Digital DNA–DNA hybridization values for MNWGS58^T^ were low when compared to *C. pseudodiphtheriticum* MNWGS56 and *C. propinquum* MNWGS51 (27.4 and 28.4%, respectively). Although there are phenotypic similarities, 85% ANI with the closest *Corynebacterium* spp. strongly supports the classification of a novel species of *Corynebacterium*, for which we propose the name *Corynebacterium drakensteinense* sp. nov., with the type strain MNWGS58^T^ (=TSD-445^T^=NCTC 15084^T^). It will be important to elucidate the role of this novel species of *Corynebacterium* in the human nasopharynx and identify additional niches for this species in future studies.

**Author Notes**

Genetic data: National Centre for Biotechnology Information (NCBI): draft genome: JAXOFP000000000 (Genome); 16S rRNA gene: OR912475 (GenBank); short reads archive: SRR27160354 (SRA). Culture collection deposits: American Type Culture Collection: TSD-445^T^; National Collection of Type Cultures: NCTC 15084^T^.

## Description of *corynebacterium drakensteinense* sp. nov.

*Corynebacterium drakensteinense* (dra.ken.stein.en’se. N.L. neut. adj. *drakensteinense*, pertaining to the Drakenstein municipality, South Africa).

Aerobic, Gram-stain positive, non-motile, 1–2 µm rod-shaped cells with typical coryneform clubbing visible by optical and electron microscopy. Optimal growth is observed on blood agar at 37 °C in aerobic conditions enriched with 5% CO_2_ for 48 h. Small colonies, ~1 mm in diameter, appear within 24 h. Mature colonies are circular and 2–4 mm in diameter with well-demarcated sharp margins. The colonies are white, and the centre is slightly raised from the growth medium. Non-CO_2_ enriched incubation yields few stunted colonies. Does not use glucose, mannose, maltose, xylose, arabinose, maltotriose or sucrose as a carbon source. Uses pyruvate as a carbon source. Metabolizes amino acids as a nitrogen and carbon source. Can reduce nitrates and is catalase positive.

The type strain is MNWGS58^T^ (=TSD-445^T^=NCTC 15084^T^), which was isolated from the nasopharynx of a healthy 30-week-old South African child in the Drakenstein municipality. The whole-genome length is 2.48 Mb with 56.9 mol% G+C content.

The annotated genome of strain MNWGS58^T^ is deposited on NCBI Genome with accession number JAXOFP000000000, and the 16S rRNA gene is deposited into NCBI GenBank with accession number OR912475.

