# Supplementary material for: Corynebacterium drakensteinense sp. nov., isolated from the nasopharynx of a healthy South African infant
Source: Int J Syst Evol Microbiol. 2026 Feb 5;76(2):007068. doi: 10.1099/ijsem.0.007068 (PMC12882079; doi:10.1099/ijsem.0.007068)
Supplement: Uncited Supplementary Material 1. [file ijsem-76-07068-s001.pdf]

Supplementary 1: Phylogenomic subtree of the genus Corynebacterium evaluated using 138 conserved genes in the phylum Actinomycetota using the GToTree tool. Trees were visualised using iTOL. Values shown are arbitrary distance values as calculated by GToTree. Bootstrap values are shown in red. Corynebacterium drakensteinense sp. nov. MNWGS58T is also shown in red. The reference genome for each Corynebacterium species was retrieved from NCBI RefSeq.

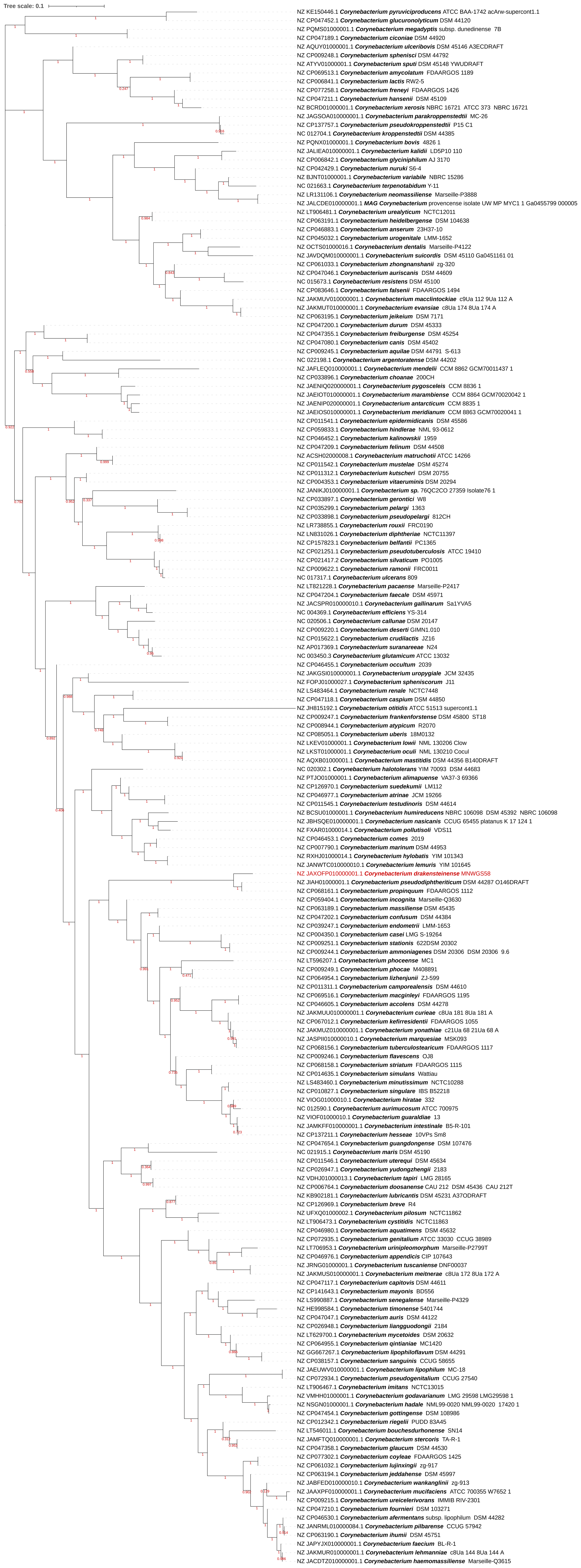

Supplementary 2: KEGG modules identified in *Corynebacterium propinquuim* MNWGS51, *Corynebacterium pseudodiphtheriticum* MNWGS56, and *Corynebacterium drakensteinense* sp. nov. MNWGS58. Green rows indicate modules that have the same level of completedness for *C. drankensteinense* sp. nov. as both comparator species (*C. propinquum* and *C. pseudodiphtheriticum*). Orange rows indicate that *C. drakensteinense* sp. nov. has a module completeness matching one but not both of the comparators. Red rows indicate *C. drakensteinense* sp. nov. had a level of module completedness that matched neither of the comparators. Horizontal lines separate KEGG

					Stepwise module completeness		
**KEGG Module**	**KEGG Module Name**	**Module class**	**Module category**	**Module subcategory**	**C. propinquum**	**C. pseudodiphtericitum**	**C. drakensteinense sp. nov.**
M00001	Glycolysis (Embden-Meyerhof pathway), glucose => pyruvate	Pathway modules	Carbohydrate metabolism	Central carbohydrate metabolism	1	1	**1**
M00002	Glycolysis, core module involving three-carbon compounds	Pathway modules	Carbohydrate metabolism	Central carbohydrate metabolism	1	1	**1**
M00003	Gluconeogenesis, oxaloacetate => fructose-6P	Pathway modules	Carbohydrate metabolism	Central carbohydrate metabolism	1	1	**1**
M00307	Pyruvate oxidation, pyruvate => acetyl-CoA	Pathway modules	Carbohydrate metabolism	Central carbohydrate metabolism	1	1	**1**
M00009	Citrate cycle (TCA cycle, Krebs cycle)	Pathway modules	Carbohydrate metabolism	Central carbohydrate metabolism	1	1	**1**
M00010	Citrate cycle, first carbon oxidation, oxaloacetate => 2-oxoglutarate	Pathway modules	Carbohydrate metabolism	Central carbohydrate metabolism	1	1	**1**
M00011	Citrate cycle, second carbon oxidation, 2-oxoglutarate => oxaloacetate	Pathway modules	Carbohydrate metabolism	Central carbohydrate metabolism	1	1	**1**
M00004	Pentose phosphate pathway (Pentose phosphate cycle)	Pathway modules	Carbohydrate metabolism	Central carbohydrate metabolism	1	1	**1**
M00006	Pentose phosphate pathway, oxidative phase, glucose 6P => ribulose 5P	Pathway modules	Carbohydrate metabolism	Central carbohydrate metabolism	1	1	**1**
M00007	Pentose phosphate pathway, non-oxidative phase, fructose 6P => ribose 5P	Pathway modules	Carbohydrate metabolism	Central carbohydrate metabolism	1	1	**1**
M00005	PRPP biosynthesis, ribose 5P => PRPP	Pathway modules	Carbohydrate metabolism	Central carbohydrate metabolism	1	1	**1**
M00008	Entner-Doudoroff pathway, glucose-6P => glyceraldehyde-3P + pyruvate	Pathway modules	Carbohydrate metabolism	Central carbohydrate metabolism	0.5	0.5	**0.5**
M00308	Semi-phosphorylative Entner-Doudoroff pathway, gluconate => glycerate-3P	Pathway modules	Carbohydrate metabolism	Central carbohydrate metabolism	0.25	0.25	**0.25**
M00014	Glucuronate pathway (uronate pathway)	Pathway modules	Carbohydrate metabolism	Other carbohydrate metabolism	0.125	0.125	**0.125**
M00632	Galactose degradation, Leloir pathway, galactose => alpha-D-glucose-1P	Pathway modules	Carbohydrate metabolism	Other carbohydrate metabolism	1	1	**1**
M00552	D-galactonate degradation, De Ley-Doudoroff pathway, D-galactonate => glycerate-3P	Pathway modules	Carbohydrate metabolism	Other carbohydrate metabolism	0.4	0.4	**0.4**
M00129	Ascorbate biosynthesis, animals, glucose-1P => ascorbate	Pathway modules	Carbohydrate metabolism	Other carbohydrate metabolism	0.285714286	0.285714286	**0.285714286**
M00114	Ascorbate biosynthesis, plants, fructose-6P => ascorbate	Pathway modules	Carbohydrate metabolism	Other carbohydrate metabolism	0.125	0.125	**0.125**
M00854	Glycogen biosynthesis, glucose-1P => glycogen/starch	Pathway modules	Carbohydrate metabolism	Other carbohydrate metabolism	0	0	**0**
M00549	Nucleotide sugar biosynthesis, glucose => UDP-glucose	Pathway modules	Carbohydrate metabolism	Other carbohydrate metabolism	0.666666667	0.666666667	**0.666666667**
M00554	Nucleotide sugar biosynthesis, galactose => UDP-galactose	Pathway modules	Carbohydrate metabolism	Other carbohydrate metabolism	1	1	**1**
M00892	UDP-N-acetyl-D-glucosamine biosynthesis, eukaryotes, glucose => UDP-GlcNAc	Pathway modules	Carbohydrate metabolism	Other carbohydrate metabolism	0.333333333	0.333333333	**0.333333333**
M00909	UDP-N-acetyl-D-glucosamine biosynthesis, prokaryotes, glucose => UDP-GlcNAc	Pathway modules	Carbohydrate metabolism	Other carbohydrate metabolism	1	1	**1**
M00012	Glyoxylate cycle	Pathway modules	Carbohydrate metabolism	Other carbohydrate metabolism	0.8	0.8	**0.8**
M00373	Ethylmalonyl pathway	Pathway modules	Carbohydrate metabolism	Other carbohydrate metabolism	0.083333333	0.083333333	**0.083333333**
M00740	Methylaspartate cycle	Pathway modules	Carbohydrate metabolism	Other carbohydrate metabolism	0.272727273	0.272727273	**0.272727273**
M00532	Photorespiration	Pathway modules	Carbohydrate metabolism	Other carbohydrate metabolism	0.2	0.2	**0.2**
M00013	Malonate semialdehyde pathway, propanoyl-CoA => acetyl-CoA	Pathway modules	Carbohydrate metabolism	Other carbohydrate metabolism	0.2	0.2	**0.4**
M00741	Propanoyl-CoA metabolism, propanoyl-CoA => succinyl-CoA	Pathway modules	Carbohydrate metabolism	Other carbohydrate metabolism	0	0	**0**
M00131	Inositol phosphate metabolism, Ins(1,3,4,5)P4 => Ins(1,3,4)P3 => myo-inositol	Pathway modules	Carbohydrate metabolism	Other carbohydrate metabolism	0.25	0.25	**0.25**
M00165	Reductive pentose phosphate cycle (Calvin cycle)	Pathway modules	Energy metabolism	Carbon fixation	0.727272727	0.727272727	**0.727272727**
M00166	Reductive pentose phosphate cycle, ribulose-5P => glyceraldehyde-3P	Pathway modules	Energy metabolism	Carbon fixation	0.5	0.5	**0.5**
M00167	Reductive pentose phosphate cycle, glyceraldehyde-3P => ribulose-5P	Pathway modules	Energy metabolism	Carbon fixation	0.857142857	0.857142857	**0.857142857**
M00173	Reductive citrate cycle (Arnon-Buchanan cycle)	Pathway modules	Energy metabolism	Carbon fixation	0.6	0.6	**0.6**
M00376	3-Hydroxypropionate bi-cycle	Pathway modules	Energy metabolism	Carbon fixation	0.153846154	0.153846154	**0.153846154**
M00375	Hydroxypropionate-hydroxybutylate cycle	Pathway modules	Energy metabolism	Carbon fixation	0.071428571	0.071428571	**0.071428571**
M00374	Dicarboxylate-hydroxybutyrate cycle	Pathway modules	Energy metabolism	Carbon fixation	0.230769231	0.230769231	**0.230769231**
M00377	Reductive acetyl-CoA pathway (Wood-Ljungdahl pathway)	Pathway modules	Energy metabolism	Carbon fixation	0.428571429	0.428571429	**0.428571429**
M00620	Incomplete reductive citrate cycle, acetyl-CoA => oxoglutarate	Pathway modules	Energy metabolism	Carbon fixation	0.142857143	0.142857143	**0.142857143**
M00346	Formaldehyde assimilation, serine pathway	Pathway modules	Energy metabolism	Methane metabolism	0.222222222	0.222222222	**0.222222222**
M00345	Formaldehyde assimilation, ribulose monophosphate pathway	Pathway modules	Energy metabolism	Methane metabolism	0.666666667	0.666666667	**0.666666667**
M00344	Formaldehyde assimilation, xylulose monophosphate pathway	Pathway modules	Energy metabolism	Methane metabolism	0.25	0.25	**0.25**
M00530	Dissimilatory nitrate reduction, nitrate => ammonia	Pathway modules	Energy metabolism	Nitrogen metabolism	0.5	0.5	**0.5**
M00529	Denitrification, nitrate => nitrogen	Pathway modules	Energy metabolism	Nitrogen metabolism	0.25	0.25	**0.25**
M00804	Complete nitrification, comammox, ammonia => nitrite => nitrate	Pathway modules	Energy metabolism	Nitrogen metabolism	0.333333333	0.333333333	**0.333333333**
M00176	Assimilatory sulfate reduction, sulfate => H2S	Pathway modules	Energy metabolism	Sulfur metabolism	0.5	0.5	**0.5**
M00149	Succinate dehydrogenase, prokaryotes	Pathway modules	Energy metabolism	ATP synthesis	0	0	**0**
M00151	Cytochrome bc1 complex respiratory unit	Pathway modules	Energy metabolism	ATP synthesis	1	1	**1**
M00154	Cytochrome c oxidase	Pathway modules	Energy metabolism	ATP synthesis	0	0	**0**
M00155	Cytochrome c oxidase, prokaryotes	Pathway modules	Energy metabolism	ATP synthesis	1	1	**1**
M00157	F-type ATPase, prokaryotes and chloroplasts	Pathway modules	Energy metabolism	ATP synthesis	1	1	**1**
M00082	Fatty acid biosynthesis, initiation	Pathway modules	Lipid metabolism	Fatty acid metabolism	0.5	0.5	**0.5**
M00083	Fatty acid biosynthesis, elongation	Pathway modules	Lipid metabolism	Fatty acid metabolism	1	1	**1**
M00874	Fatty acid biosynthesis in mitochondria, fungi	Pathway modules	Lipid metabolism	Fatty acid metabolism	0.166666667	0.166666667	**0.166666667**
M00086	beta-Oxidation, acyl-CoA synthesis	Pathway modules	Lipid metabolism	Fatty acid metabolism	1	1	**1**
M00087	beta-Oxidation	Pathway modules	Lipid metabolism	Fatty acid metabolism	0.666666667	0.666666667	**0.666666667**
M00861	beta-Oxidation, peroxisome, VLCFA	Pathway modules	Lipid metabolism	Fatty acid metabolism	0.333333333	0.333333333	**0.333333333**
M00088	Ketone body biosynthesis, acetyl-CoA => acetoacetate/3-hydroxybutyrate/acetone	Pathway modules	Lipid metabolism	Lipid metabolism	0.2	0.2	**0.2**
M00089	Triacylglycerol biosynthesis	Pathway modules	Lipid metabolism	Lipid metabolism	0.25	0.25	**0.25**
M00098	Acylglycerol degradation	Pathway modules	Lipid metabolism	Lipid metabolism	0.5	0.5	**0.5**
M00093	Phosphatidylethanolamine (PE) biosynthesis, PA => PS => PE	Pathway modules	Lipid metabolism	Lipid metabolism	0.333333333	0.333333333	**0.333333333**

M00113	Jasmonic acid biosynthesis	Pathway modules	Lipid metabolism	Lipid metabolism	0.111111111	0.111111111	**0.111111111**
M00048	De novo purine biosynthesis, PRPP + glutamine => IMP	Pathway modules	Nucleotide metabolism	Purine metabolism	1	1	**1**
M00049	Adenine ribonucleotide biosynthesis, IMP => ADP,ATP	Pathway modules	Nucleotide metabolism	Purine metabolism	1	1	**1**
M00050	Guanine ribonucleotide biosynthesis, IMP => GDP,GTP	Pathway modules	Nucleotide metabolism	Purine metabolism	1	1	**1**
M00053	Deoxyribonucleotide biosynthesis, ADP/GDP/CDP/UDP => dATP/dGTP/dCTP/dUTP	Pathway modules	Nucleotide metabolism	Purine metabolism	1	1	**1**
M00958	Adenine ribonucleotide degradation, AMP => Urate	Pathway modules	Nucleotide metabolism	Purine metabolism	0.333333333	0.333333333	**0.333333333**
M00959	Guanine ribonucleotide degradation, GMP => Urate	Pathway modules	Nucleotide metabolism	Purine metabolism	0.25	0.25	**0.25**
M00051	De novo pyrimidine biosynthesis, glutamine (+ PRPP) => UMP	Pathway modules	Nucleotide metabolism	Pyrimidine metabolism	0.666666667	0.666666667	**0.666666667**
M00052	Pyrimidine ribonucleotide biosynthesis, UMP => UDP/UTP,CDP/CTP	Pathway modules	Nucleotide metabolism	Pyrimidine metabolism	0.666666667	0.666666667	**0.666666667**
M00938	Pyrimidine deoxyribonucleotide biosynthesis, UDP => dTTP	Pathway modules	Nucleotide metabolism	Pyrimidine metabolism	1	1	**1**
M00046	Pyrimidine degradation, uracil => beta-alanine, thymine => 3-aminoisobutanoate	Pathway modules	Nucleotide metabolism	Pyrimidine metabolism	0.333333333	0	**0.333333333**
M00020	Serine biosynthesis, glycerate-3P => serine	Pathway modules	Amino acid metabolism	Serine and threonine metabolism	1	1	**1**
M00018	Threonine biosynthesis, aspartate => homoserine => threonine	Pathway modules	Amino acid metabolism	Serine and threonine metabolism	1	1	**1**
M00621	Glycine cleavage system	Pathway modules	Amino acid metabolism	Serine and threonine metabolism	0.333333333	0.333333333	**0.333333333**
M00555	Betaine biosynthesis, choline => betaine	Pathway modules	Amino acid metabolism	Serine and threonine metabolism	0	0	**0**
M00033	Ectoine biosynthesis, aspartate => ectoine	Pathway modules	Amino acid metabolism	Serine and threonine metabolism	0.4	0.4	**0.6**
M00021	Cysteine biosynthesis, serine => cysteine	Pathway modules	Amino acid metabolism	Cysteine and methionine metabolism	1	1	**1**
M00609	Cysteine biosynthesis, methionine => cysteine	Pathway modules	Amino acid metabolism	Cysteine and methionine metabolism	0.166666667	0.166666667	**0.166666667**
M00017	Methionine biosynthesis, aspartate => homoserine => methionine	Pathway modules	Amino acid metabolism	Cysteine and methionine metabolism	1	1	**1**
M00034	Methionine salvage pathway	Pathway modules	Amino acid metabolism	Cysteine and methionine metabolism	0.25	0.25	**0.25**
M00035	Methionine degradation	Pathway modules	Amino acid metabolism	Cysteine and methionine metabolism	0.25	0.25	**0.5**
M00368	Ethylene biosynthesis, methionine => ethylene	Pathway modules	Amino acid metabolism	Cysteine and methionine metabolism	0.333333333	0.333333333	**0.333333333**
M00019	Valine/isoleucine biosynthesis, pyruvate => valine / 2-oxobutanoate => isoleucine	Pathway modules	Amino acid metabolism	Branched-chain amino acid metabolism	1	1	**1**
M00535	Isoleucine biosynthesis, pyruvate => 2-oxobutanoate	Pathway modules	Amino acid metabolism	Branched-chain amino acid metabolism	0.666666667	0.666666667	**0.666666667**
M00570	Isoleucine biosynthesis, threonine => 2-oxobutanoate => isoleucine	Pathway modules	Amino acid metabolism	Branched-chain amino acid metabolism	1	1	**1**
M00432	Leucine biosynthesis, 2-oxoisovalerate => 2-oxoisocaproate	Pathway modules	Amino acid metabolism	Branched-chain amino acid metabolism	1	1	**1**
M00036	Leucine degradation, leucine => acetoacetate + acetyl-CoA	Pathway modules	Amino acid metabolism	Branched-chain amino acid metabolism	0.166666667	0.166666667	**0.166666667**
M00016	Lysine biosynthesis, succinyl-DAP pathway, aspartate => lysine	Pathway modules	Amino acid metabolism	Lysine metabolism	1	1	**1**
M00525	Lysine biosynthesis, acetyl-DAP pathway, aspartate => lysine	Pathway modules	Amino acid metabolism	Lysine metabolism	0.777777778	0.777777778	**0.666666667**
M00526	Lysine biosynthesis, DAP dehydrogenase pathway, aspartate => lysine	Pathway modules	Amino acid metabolism	Lysine metabolism	0.833333333	0.833333333	**0.833333333**
M00527	Lysine biosynthesis, DAP aminotransferase pathway, aspartate => lysine	Pathway modules	Amino acid metabolism	Lysine metabolism	0.857142857	0.857142857	**0.857142857**
M00032	Lysine degradation, lysine => saccharopine => acetoacetyl-CoA	Pathway modules	Amino acid metabolism	Lysine metabolism	0.166666667	0.166666667	**0.166666667**
M00956	Lysine degradation, bacteria, L-lysine => succinate	Pathway modules	Amino acid metabolism	Lysine metabolism	0.285714286	0.142857143	**0.285714286**
M00957	Lysine degradation, bacteria, L-lysine => glutarate => succinate/acetyl-CoA	Pathway modules	Amino acid metabolism	Lysine metabolism	0.4	0.2	**0.4**
M00028	Ornithine biosynthesis, glutamate => ornithine	Pathway modules	Amino acid metabolism	Arginine and proline metabolism	1	1	**1**
M00844	Arginine biosynthesis, ornithine => arginine	Pathway modules	Amino acid metabolism	Arginine and proline metabolism	1	1	**1**
M00845	Arginine biosynthesis, glutamate => acetylcitrulline => arginine	Pathway modules	Amino acid metabolism	Arginine and proline metabolism	0.571428571	0.571428571	**0.571428571**
M00029	Urea cycle	Pathway modules	Amino acid metabolism	Arginine and proline metabolism	0.6	0.6	**0.6**
M00015	Proline biosynthesis, glutamate => proline	Pathway modules	Amino acid metabolism	Arginine and proline metabolism	1	1	**1**
M00970	Proline degradation, proline => glutamate	Pathway modules	Amino acid metabolism	Arginine and proline metabolism	1	1	**1**
M00972	Proline metabolism	Pathway modules	Amino acid metabolism	Arginine and proline metabolism	0.333333333	0.333333333	**0.333333333**
M00026	Histidine biosynthesis, PRPP => histidine	Pathway modules	Amino acid metabolism	Histidine metabolism	1	1	**1**
M00045	Histidine degradation, histidine => N-formiminoglutamate => glutamate	Pathway modules	Amino acid metabolism	Histidine metabolism	1	1	**1**
M00022	Shikimate pathway, phosphoenolpyruvate + erythrose-4P => chorismate	Pathway modules	Amino acid metabolism	Aromatic amino acid metabolism	1	1	**1**
M00023	Tryptophan biosynthesis, chorismate => tryptophan	Pathway modules	Amino acid metabolism	Aromatic amino acid metabolism	1	1	**1**
M00024	Phenylalanine biosynthesis, chorismate => phenylpyruvate => phenylalanine	Pathway modules	Amino acid metabolism	Aromatic amino acid metabolism	0.5	0.5	**0.5**
M00025	Tyrosine biosynthesis, chorismate => HPP => tyrosine	Pathway modules	Amino acid metabolism	Aromatic amino acid metabolism	0.5	0.5	**0.5**
M00533	Homoprotocatechuate degradation, homoprotocatechuate => 2-oxohept-3-enedioate	Pathway modules	Amino acid metabolism	Aromatic amino acid metabolism	0.5	0	**0.5**
M00027	GABA (gamma-Aminobutyrate) shunt	Pathway modules	Amino acid metabolism	Other amino acid metabolism	0.666666667	0.333333333	**0.666666667**
M00872	O-glycan biosynthesis, mannose type (core M3)	Pathway modules	Glycan metabolism	Glycan biosynthesis	0.1	0.1	**0.1**
M00127	Thiamine biosynthesis, prokaryotes, AIR (+ DXP/tyrosine) => TMP/TPP	Pathway modules	Metabolism of cofactors and vitamins	Cofactor and vitamin metabolism	0.571428571	0.571428571	**0.571428571**
M00895	Thiamine biosynthesis, prokaryotes, AIR (+ DXP/glycine) => TMP/TPP	Pathway modules	Metabolism of cofactors and vitamins	Cofactor and vitamin metabolism	0.666666667	0.666666667	**0.666666667**
M00896	Thiamine biosynthesis, archaea, AIR (+ NAD+) => TMP/TPP	Pathway modules	Metabolism of cofactors and vitamins	Cofactor and vitamin metabolism	0.75	0.75	**0.75**
M00897	Thiamine biosynthesis, plants, AIR (+ NAD+) => TMP/thiamine/TPP	Pathway modules	Metabolism of cofactors and vitamins	Cofactor and vitamin metabolism	0.2	0.2	**0.2**
M00899	Thiamine salvage pathway, HMP/HET => TMP	Pathway modules	Metabolism of cofactors and vitamins	Cofactor and vitamin metabolism	0.5	0.5	**0.5**
M00125	Riboflavin biosynthesis, plants and bacteria, GTP => riboflavin/FMN/FAD	Pathway modules	Metabolism of cofactors and vitamins	Cofactor and vitamin metabolism	1	1	**1**
M00911	Riboflavin biosynthesis, fungi, GTP => riboflavin/FMN/FAD	Pathway modules	Metabolism of cofactors and vitamins	Cofactor and vitamin metabolism	0.222222222	0.222222222	**0.222222222**
M00124	Pyridoxal-P biosynthesis, erythrose-4P => pyridoxal-P	Pathway modules	Metabolism of cofactors and vitamins	Cofactor and vitamin metabolism	0.166666667	0.166666667	**0.166666667**
M00916	Pyridoxal-P biosynthesis, R5P + glyceraldehyde-3P + glutamine => pyridoxal-P	Pathway modules	Metabolism of cofactors and vitamins	Cofactor and vitamin metabolism	0	0	**0**
M00115	NAD biosynthesis, aspartate => quinolinate => NAD	Pathway modules	Metabolism of cofactors and vitamins	Cofactor and vitamin metabolism	0.8	0.8	**0.8**
M00912	NAD biosynthesis, tryptophan => quinolinate => NAD	Pathway modules	Metabolism of cofactors and vitamins	Cofactor and vitamin metabolism	0.375	0.375	**0.375**
M00119	Pantothenate biosynthesis, valine/L-aspartate => pantothenate	Pathway modules	Metabolism of cofactors and vitamins	Cofactor and vitamin metabolism	0.8	0.8	**0.8**
M00913	Pantothenate biosynthesis, 2-oxoisovalerate/spermine => pantothenate	Pathway modules	Metabolism of cofactors and vitamins	Cofactor and vitamin metabolism	0.4	0.4	**0.4**
M00120	Coenzyme A biosynthesis, pantothenate => CoA	Pathway modules	Metabolism of cofactors and vitamins	Cofactor and vitamin metabolism	1	1	**1**
M00914	Coenzyme A biosynthesis, archaea, 2-oxoisovalerate => 4-phosphopantoate => CoA	Pathway modules	Metabolism of cofactors and vitamins	Cofactor and vitamin metabolism	0.285714286	0.285714286	**0.285714286**

M00572	Pimeloyl-ACP biosynthesis, BioC-BioH pathway, malonyl-ACP => pimeloyl-ACP	Pathway modules	Metabolism of cofactors and vitamins	Cofactor and vitamin metabolism	0.166666667	0.166666667	**0.166666667**
M00123	Biotin biosynthesis, pimeloyl-ACP/CoA => biotin	Pathway modules	Metabolism of cofactors and vitamins	Cofactor and vitamin metabolism	1	1	**0.666666667**
M00950	Biotin biosynthesis, BioU pathway, pimeloyl-ACP/CoA => biotin	Pathway modules	Metabolism of cofactors and vitamins	Cofactor and vitamin metabolism	0.75	0.75	**0.5**
M00573	Biotin biosynthesis, BioI pathway, long-chain-acyl-ACP => pimeloyl-ACP => biotin	Pathway modules	Metabolism of cofactors and vitamins	Cofactor and vitamin metabolism	0.6	0.6	**0.4**
M00577	Biotin biosynthesis, BioW pathway, pimelate => pimeloyl-CoA => biotin	Pathway modules	Metabolism of cofactors and vitamins	Cofactor and vitamin metabolism	1	1	**0.8**
M00881	Lipoic acid biosynthesis, plants and bacteria, octanoyl-ACP => dihydrolipoyl-E2/H	Pathway modules	Metabolism of cofactors and vitamins	Cofactor and vitamin metabolism	1	1	**1**
M00882	Lipoic acid biosynthesis, eukaryotes, octanoyl-ACP => dihydrolipoyl-H	Pathway modules	Metabolism of cofactors and vitamins	Cofactor and vitamin metabolism	0.5	0.5	**0.5**
M00883	Lipoic acid biosynthesis, animals and bacteria, octanoyl-ACP => dihydrolipoyl-H => dihydrolipoyl-	Pathway modules	Metabolism of cofactors and vitamins	Cofactor and vitamin metabolism	0.333333333	0.333333333	**0.333333333**
M00884	Lipoic acid biosynthesis, octanoyl-CoA => dihydrolipoyl-E2	Pathway modules	Metabolism of cofactors and vitamins	Cofactor and vitamin metabolism	0.5	0.5	**0.5**
M00126	Tetrahydrofolate biosynthesis, GTP => THF	Pathway modules	Metabolism of cofactors and vitamins	Cofactor and vitamin metabolism	0.8	0.8	**1**
M00840	Tetrahydrofolate biosynthesis, mediated by ribA and trpF, GTP => THF	Pathway modules	Metabolism of cofactors and vitamins	Cofactor and vitamin metabolism	0.428571429	0.428571429	**0.428571429**
M00841	Tetrahydrofolate biosynthesis, mediated by PTPS, GTP => THF	Pathway modules	Metabolism of cofactors and vitamins	Cofactor and vitamin metabolism	0.4	0.4	**0.4**
M00842	Tetrahydrobiopterin biosynthesis, GTP => BH4	Pathway modules	Metabolism of cofactors and vitamins	Cofactor and vitamin metabolism	0.333333333	0.333333333	**0.333333333**
M00843	L-threo-Tetrahydrobiopterin biosynthesis, GTP => L-threo-BH4	Pathway modules	Metabolism of cofactors and vitamins	Cofactor and vitamin metabolism	0.333333333	0.333333333	**0.333333333**
M00880	Molybdenum cofactor biosynthesis, GTP => molybdenum cofactor	Pathway modules	Metabolism of cofactors and vitamins	Cofactor and vitamin metabolism	0.666666667	0.666666667	**1**
M00140	C1-unit interconversion, prokaryotes	Pathway modules	Metabolism of cofactors and vitamins	Cofactor and vitamin metabolism	1	1	**1**
M00141	C1-unit interconversion, eukaryotes	Pathway modules	Metabolism of cofactors and vitamins	Cofactor and vitamin metabolism	0.5	0.5	**0.5**
M00846	Siroheme biosynthesis, glutamyl-tRNA => siroheme	Pathway modules	Metabolism of cofactors and vitamins	Cofactor and vitamin metabolism	0.833333333	0.833333333	**0.833333333**
M00868	Heme biosynthesis, animals and fungi, glycine => heme	Pathway modules	Metabolism of cofactors and vitamins	Cofactor and vitamin metabolism	0.75	0.75	**0.75**
M00121	Heme biosynthesis, plants and bacteria, glutamate => heme	Pathway modules	Metabolism of cofactors and vitamins	Cofactor and vitamin metabolism	1	1	**1**
M00926	Heme biosynthesis, bacteria, glutamyl-tRNA => coproporphyrin III => heme	Pathway modules	Metabolism of cofactors and vitamins	Cofactor and vitamin metabolism	1	1	**1**
M00924	Cobalamin biosynthesis, anaerobic, uroporphyrinogen III => sirohydrochlorin => cobyrinate a,c-d	Pathway modules	Metabolism of cofactors and vitamins	Cofactor and vitamin metabolism	0.090909091	0.090909091	**0.090909091**
M00925	Cobalamin biosynthesis, aerobic, uroporphyrinogen III => precorrin 2 => cobyrinate a,c-diamide	Pathway modules	Metabolism of cofactors and vitamins	Cofactor and vitamin metabolism	0.090909091	0.090909091	**0.090909091**
M00122	Cobalamin biosynthesis, cobyrinate a,c-diamide => cobalamin	Pathway modules	Metabolism of cofactors and vitamins	Cofactor and vitamin metabolism	0.285714286	0.285714286	**0.285714286**
M00117	Ubiquinone biosynthesis, prokaryotes, chorismate (+ polyprenyl-PP) => ubiquinol	Pathway modules	Metabolism of cofactors and vitamins	Cofactor and vitamin metabolism	0.111111111	0.111111111	**0.111111111**
M00116	Menaquinone biosynthesis, chorismate (+ polyprenyl-PP) => menaquinol	Pathway modules	Metabolism of cofactors and vitamins	Cofactor and vitamin metabolism	0.777777778	0.777777778	**0.666666667**
M00932	Phylloquinone biosynthesis, chorismate (+ phytyl-PP) => phylloquinol	Pathway modules	Metabolism of cofactors and vitamins	Cofactor and vitamin metabolism	0.428571429	0.428571429	**0.285714286**
M00095	C5 isoprenoid biosynthesis, mevalonate pathway	Pathway modules	Biosynthesis of terpenoids and polyketides	Terpenoid backbone biosynthesis	0.142857143	0.142857143	**0.142857143**
M00849	C5 isoprenoid biosynthesis, mevalonate pathway, archaea	Pathway modules	Biosynthesis of terpenoids and polyketides	Terpenoid backbone biosynthesis	0.166666667	0.166666667	**0.166666667**
M00096	C5 isoprenoid biosynthesis, non-mevalonate pathway	Pathway modules	Biosynthesis of terpenoids and polyketides	Terpenoid backbone biosynthesis	0.875	0.875	**0.875**
M00364	C10-C20 isoprenoid biosynthesis, bacteria	Pathway modules	Biosynthesis of terpenoids and polyketides	Terpenoid backbone biosynthesis	0.5	0.5	**0.5**
M00365	C10-C20 isoprenoid biosynthesis, archaea	Pathway modules	Biosynthesis of terpenoids and polyketides	Terpenoid backbone biosynthesis	0.5	0.5	**0.5**
M00097	beta-Carotene biosynthesis, GGAP => beta-carotene	Pathway modules	Biosynthesis of terpenoids and polyketides	Plant terpenoid biosynthesis	0.166666667	0.166666667	**0.166666667**
M00780	Tetracycline/oxytetracycline biosynthesis, pretetramide => tetracycline/oxytetracycline	Pathway modules	Biosynthesis of terpenoids and polyketides	Type II polyketide biosynthesis	0.142857143	0.142857143	**0.142857143**
M00823	Chlortetracycline biosynthesis, pretetramide => chlortetracycline	Pathway modules	Biosynthesis of terpenoids and polyketides	Type II polyketide biosynthesis	0.125	0.125	**0.125**
M00793	dTDP-L-rhamnose biosynthesis	Pathway modules	Biosynthesis of terpenoids and polyketides	Polyketide sugar unit biosynthesis	1	1	**0.666666667**
M00039	Monolignol biosynthesis, phenylalanine/tyrosine => monolignol	Pathway modules	Biosynthesis of other secondary metabolites	Biosynthesis of phytochemical compounds	0.1	0.1	**0**
M00137	Flavanone biosynthesis, phenylalanine => naringenin	Pathway modules	Biosynthesis of other secondary metabolites	Biosynthesis of phytochemical compounds	0.2	0.2	**0**
M00953	Mugineic acid biosynthesis, methionine => 3-epihydroxymugineic acid	Pathway modules	Biosynthesis of other secondary metabolites	Biosynthesis of phytochemical compounds	0.2	0.2	**0.2**
M00848	Aurachin biosynthesis, anthranilate => aurachin A	Pathway modules	Biosynthesis of other secondary metabolites	Biosynthesis of other antibiotics	0	0	**0**
M00951	Cremeomycin biosynthesis, aspartate/3,4-AHBA => cremeomycin	Pathway modules	Biosynthesis of other secondary metabolites	Biosynthesis of other antibiotics	0.2	0.2	**0.2**
M00835	Pyocyanine biosynthesis, chorismate => pyocyanine	Pathway modules	Biosynthesis of other secondary metabolites	Biosynthesis of other bacterial compounds	0.142857143	0.142857143	**0.142857143**
M00921	Cyclooctatin biosynthesis, dimethylallyl-PP + isopentenyl-PP => cyclooctatin	Pathway modules	Biosynthesis of other secondary metabolites	Biosynthesis of other bacterial compounds	0.25	0.25	**0.25**
M00876	Staphyloferrin A biosynthesis, L-ornithine => staphyloferrin A	Pathway modules	Biosynthesis of other secondary metabolites	Biosynthesis of other bacterial compounds	0	0.666666667	**0**
M00918	Aerobactin biosynthesis, lysine => aerobactin	Pathway modules	Biosynthesis of other secondary metabolites	Biosynthesis of other bacterial compounds	0.25	0	**0.25**
M00569	Catechol meta-cleavage, catechol => acetyl-CoA / 4-methylcatechol => propanoyl-CoA	Pathway modules	Xenobiotics biodegradation	Aromatics degradation	0.2	0	**0.2**
M00878	Phenylacetate degradation, phenylaxetate => acetyl-CoA/succinyl-CoA	Pathway modules	Xenobiotics biodegradation	Aromatics degradation	0.571428571	0.142857143	**0.571428571**
M00651	Vancomycin resistance, D-Ala-D-Lac type	Signature modules	Gene set	Drug resistance	0	0	**0**
M00725	Cationic antimicrobial peptide (CAMP) resistance, dltABCD operon	Signature modules	Gene set	Drug resistance	0	0	**0**
M00714	Multidrug resistance, efflux pump QacA	Signature modules	Gene set	Drug resistance	0.5	0.5	**0.5**
M00611	Oxygenic photosynthesis in plants and cyanobacteria	Signature modules	Module set	Metabolic capacity	0	0	**0**
M00612	Anoxygenic photosynthesis in purple bacteria	Signature modules	Module set	Metabolic capacity	0	0	**0**
M00613	Anoxygenic photosynthesis in green nonsulfur bacteria	Signature modules	Module set	Metabolic capacity	0	0	**0**
M00614	Anoxygenic photosynthesis in green sulfur bacteria	Signature modules	Module set	Metabolic capacity	0	0	**0**
M00618	Acetogen	Signature modules	Module set	Metabolic capacity	0	0	**0**
M00615	Nitrate assimilation	Signature modules	Module set	Metabolic capacity	0.5	0.5	**0.5**
M00616	Sulfate-sulfur assimilation	Signature modules	Module set	Metabolic capacity	0	0	**0**
